# Asthma and Allergic Rhinitis Correlation in Palm Tree Workers of Jahrom City in 2016

**Published:** 2017-05

**Authors:** Mohammad Amin Farahmand Fard, Narges Khanjani, Aliasghar Arabi Mianroodi, Ahad Ashrafi Asgarabad

**Affiliations:** 1 *Department of Epidemiology, School of Public Health, Kerman University of Medical Sciences, Kerman, Iran.*; 2 *Environmental Health Engineering Research Center, Kerman University of Medical Sciences, Kerman, Iran. *; 3 *Department of Otorhinolaryngology, Faculty of Medicine, Kerman University of Medical Sciences, Kerman, Iran.*; 4 *Department of Public Health, School of Public Health, Bam University of Medical Sciences, Bam, Iran*

**Keywords:** Allergic rhinitis, Asthma, date garden, Iran, Jahrom

## Abstract

**Introduction:**

Allergic rhinitis and asthma can be related to occupation. The present study aimed to investigate the correlation between asthma or allergic rhinitis and employment in the palm tree gardens of Jahrom, Iran.

**Materials and Methods:**

This was a cross-sectional study including 50 palm tree garden workers and a control group of 50 office employees. Data collection included demographics, as well as standard International Study of Asthma and Allergies in Childhood (ISAAC) and A New Symptom-Based Questionnaire for Predicting the Presence of Asthma (ASQ) questionnaires. Data were analyzed using SPSS22. Descriptive statistics, chi-square test, t-test, and logistics regression were used to analyze data.

**Results:**

The correlation between asthma and occupation was significant ( P=0.046); and asthma prevalence was higher in palm tree garden workers. However, no relationship was observed between age, duration of employment, smoking cigarettes, hookah, or opium addiction with asthma. Furthermore, in this study, no significant relation was observed between the prevalence of asthma and contact with dust, contact with pets’ skin and hair, family history of asthma, or the use of perfume and air freshener. The symptoms of allergic rhinitis (including sneezing, runny nose, and blocked nose) were significantly greater in palm tree garden workers (P=0.038). These symptoms in both workers and office employees were higher in spring.

**Conclusion:**

In our study, allergic rhinitis and asthma were more common in palm tree garden workers than in the general population. According to our study, people working in this occupation should take necessary precautions.

## Introduction

Asthma is a clinical syndrome that causes inflammation, irritation or tightness (spasticity) of the airways. Dyspnea, wheezing and cough are consequences of this spasm. Recently, the epidemiology of asthma and allergic disease has attracted much attention and has assumed considerable importance due to the increased prevalence and severity of these conditions, as well as their social and economic impact on public health services. Asthma is a common chronic disease globally, and it is estimated that currently there are 300 million asthmatics in the world. It is predicted that the world's population of asthmatic patients will increase by 100 million people by 2025, largely due to increasing urbanization. In the 2003 Global Burden of Asthma Report, the prevalence of asthma in the total population in Iran was estimated to be 5.5% (1). Allergic rhinitis is also known to be prevalent in different parts of the world, with prevalence rates of 10–40% across different age groups. Classic clinical symptoms of allergic rhinitis include sneezing, itching, congestion, and rhinorrhea (1).

A large number of people have asthma and allergic rhinitis concurrently. Approximately 30% of patients with allergic rhinitis develop asthma, while approximately 60% of patients with asthma develop allergic rhinitis. These two diseases have a common pathophysiology and their risk and exacerbation factors are common. Contact with airborne allergens inside and outside the home leads to the development and exacerbation of problems in patients with asthma or allergic rhinitis, or both. These allergens include dust mites, pollens, trees, plants, fungi, and animal skin (2).

Studies on the correlation of agriculture and the development of asthma and allergic rhinitis are scarce. In our study, we aimed to investigate the correlation between asthma and allergic rhinitis and employment in the palm tree gardens of Jahrom, a major date growing town in Iran.

## Materials and Methods

This study was a cross-sectional study conducted in May and June 2016. Fifty male palm tree garden workers and 50 male office employees participated in the study. The members of the control group were office employees in the same town, but did not work in the same gardens and did not have constant contact with the trees. The two groups were matched for age.

The necessary inquiries were made to the Palm and Citrus Tree Authority of Jahrom and permission was granted to conduct this study. Then, a list of the palm tree gardens and their workers was requested from the town authorities. Inclusion criteria for the workers group included being employed in the date gardens of Jahrom for at least 1 year, such that it was their full-time job and they had no other employment. Inclusion criteria for the control group included not being employed in the date gardens and not dealing with these trees routinely.

Because no prior studies had been performed on this topic, we performed a pilot study in 20 workers and 20 controls and asked them to complete the questionnaire. After estimating the prevalence of the diseases in both groups, the sample size for the current study was calculated, and 50 people in each group were recruited into the study.

In order to complete the questionnaire, researchers visited the gardens and offices and asked people for their consent to participate in this study. Then, three questionnaires for asthma, allergic rhinitis, and demographic information were completed by all participants. The questionnaires are described below.

The ASQ (A New Symptom - Based Questio- nnaire for Predicting the Presence of Asthma) questionnaire consists of six scored questions, including shortness of breath, coughing, wheezing, and dyspnea. In this questionnaire, patients with a score of 4 or more are considered asthmatic. The ISAAC (International Study of Asthma and Allergies in Childhood) allergy questionnaire includes six yes/no questions in which the existence of symptoms in each person is questioned (3-5).

The demographic questionnaire consisted of four parts, including demographic information, positive history of asthma and allergic rhinitis in the family, smoking status, and being in contact with asthma risk factors. The ISAAC allergic rhinitis questionnaire has been used in many studies in Iran and its validity and reliability has been proved (3-5). In order to determine the validity of the ASQ questionnaire in Farsi, expert opinion was used. Reliability was tested by test-retest. Internal validity (Cronbach's alpha) was 0.73. In order to examine the crude association between asthma and allergic rhinitis with occupation and other variables, the chi-square and the t-test were used. In order to investigate the relationship between employment in the palm tree gardens and asthma and allergic rhinitis, adjusted for confounding factors, logistics regression was used. In all tests, a 95% confidence level was considered. SPSS 22 software was used for statistical analysis.

The Ethics in Research Committee of Kerman University of Medical Sciences approved the study protocol (Ethics Approval No IR. KMU. REC. 1395.194).

## Results

One hundred people, including 50 male palm tree workers and 50 male office employees, participated in this study. In Table 1, the demography of the population is presented and compared. According to Table 2, the age and length of employment were mainly the same in the two groups. However, the rate of asthma was different between the two groups (P=0.046; Table.3). No relationship was seen between smoking cigarettes, hookah and drug addiction with asthma. Also, no significant relationship was observed between the prevalence of asthma and dust, contact with pets’ skin and hair, family history of asthma, or the use of perfume and air fresheners. Symptoms of allergic rhinitis such as sneezing, runny nose, or blocked nose were present in more palm tree workers, and the difference between the two groups was significant (P=0.038; Table 4). Symptoms in both workers and office employees were more common in spring. Multivariate logistic analysis of the factors related to asthma and allergy are presented in Table 5. There was no significant correlation between asthma and the variables, expect for an increased risk in people with opium drug abuse. In addition, a significant increase in allergic rhinitis was seen in people with a history of allergic rhinitis in their family.

**Table 1:** Demographic variables in the study population

**Variable**	**Palm tree garden workers ** number (percentage)	**Office employees** number (percentage)	**P-value**
Marital status			
Married	40 (80)	40 (80)	0.59
Unmarried	10 (20)	10 (20)	
Total	50 (100)	50 (100)	
Education			
8th grade and lower	43 (86)	3 (6)	0.0001
Higher than 8th grade	7 (14)	47 (94)	
Total	50 (100)	50 (100)	
Smoking			
Yes	17 (34)	7 (14)	0.019
No	33 (66)	43 (86)	
Total	50 (100)	50 (100)	
Hookah smoking			
Yes	8 (16)	4 (8)	0.26
No	42 (84)	46 (92)	
Total	50 (100)	50 (100)	
Opium drug abuse			
Yes	5 (10)	0 (0)	0.02
No	45 (90)	50 (100)	
Total	50 (100)	50 (100)	
Exposure to second-hand smoking from cigarettes			
Yes	24 (48)	21 (42)	0.54
No	26 (52)	29 (58)	
Total	50 (100)	50 (100)	
Exposure to second-hand smoking from hookah			
Yes	8 (16)	7 (14)	0.77
No	42 (84)	43 (86)	
Total	50 (100)	50 (100)	
Exposure to second-hand from opium drug abuse			
Yes	11 (22)	9 (18)	0.61
No	39 (78)	41 (82)	
Total	50 (100)	50 (100)	

**Table 2:** Comparison of age and duration of employment

**Variable**	**Palm tree garden worker ** **Mean (SD)**	**Employee ** **Mean (SD)**	**P-value**
Age (years)	38.98 (11.91)	37.96 (8.38)	0.81
Duration of employment (years)	15.38 (11.8)	13.34 (8.12)	0.67

**Table 3:** Prevalence of asthma in the two groups

**Variable**	**Asthma**	**Present**	**No asthma**	**Total**	**P-value**
		**Frequency ** **(%)**	**Frequency** ** (%)**	**Frequency ** **(%)**	
Occupation	Palm tree garden workers	8 (16)	42 (84)	50 (100)	0.046
	Office employee	2 (4)	48 (96)	50 (100)	
	Total	10 (10)	90 (90)	50 (100)	

**Table 4:** The frequency and prevalence of allergic rhinitis symptoms based on ISSAC

Variable Symptoms of allergic rhinitis	Palm tree garden workers number (percentage)	Office employees number (percentage)	P-value
1. Have you ever had a problem with sneezing, or a runny, or a blocked nose when you DID NOT have a cold or the flu?			0.038
Yes	17 (34)	8 (16)	
No	33 (66)	42 (84)	
Total	50 (100)	50 (100)	
2. In the past 12 months, have you had a problem with sneezing, or a runny, or a blocked nose when you DID NOT have a cold or the flu?			1
Yes	17 (100)	8 (100)	
No	-	-	
Total	17 (100)	8 (100)	
3. In the past 12 months, has this nose problem been accompanied by itchy-watery eyes?			0.66
Yes	14 (82.4)	6 (75)	
No	3 (17.6)	2 (25)	
Total	17 (100)	8 (100)	
4. In the past 12 months, what seasons did this nose problem occur?			0.17
Spring	16 (94.1)	6 (75)	
Summer	1 (5.9)	2 (25)	
Total	17 (100)	8 (100)	
5. In the past 12 months, how much did this nose problem interfere with your daily activities?			0.18
And low	4 (23.5)	4 (50)	
Medium and high	13 (76.5)	4 (50)	
Total	17 (100)	8 (100)	
6. Have you ever had hay fever?			0.74
Yes	6 (12)	5 (10)	
No	44 (88)	45 (90)	
Total	50 (100)	50 (100)	

**Table 5:** Multivariate logistic regression analysis

**Variable**	**Asthma**		**Allergic rhinitis symptoms**	
	**OR (95% CI)** **Crude**	**OR (95% CI)** **Adjusted**	**OR (95% CI)** **Crude**	**OR (95% CI)** **Adjusted**
Education				
8th grade and lower Higher than 8th grade	1 -	1 5.47 (1.1-22.74)	1 1.06 (0.19-5.93)	1 2.12 (0.84-5.35)
Marital status				
Married Unmarried	1 -	1 -	1 0.85 (0.18-4.03)	1 1 (0.32-3.1)
Family history of asthma				
Yes No	1 -	1 2.56 (0.46-14.18)	1 1.78 (0.22-13.97)	1 1.32 (0.31-5.65)
Family history of Allergic rhinitis				
Yes No	1 0.06 (0.001-5.76)	1 0.72 (0.08-6.22)	1 7.69 (1.57-37.65)	1 6.58 (1.91-22.69)
Smoking				
Yes No	1 1.71 (0.11-24.63)	1 3.73 (0.97-14.25)	1 2.6 (0.5-13.34)	1 1.32 (4.75-3.7)
Exposure to second-hand smoking from cigarettes				
Yes No	1 0.05 (0.002-1.74)	1 1.96 (0.51-7.43)	1 0.22 (0.04-1.13)	1 0.76 (0.3-1.91)
Hookah smoking				
Yes No	1 -	1 3.85 (0.84-17.5)	1 3.68 (0.66-20.52)	1 2.42 (0.69-8.48)
Exposure to second-hand smoking from hookah				
Yes No	1 -	1 2.78 (0.63-12.27)	1 0.95 (0.16-5.47)	1 1.62 (0.49-5.31)
Opium drug abuse				
Yes No	1 0.17 (0.004-7.14)	1 2.38 (0.24-23.7)	1 1.23 (0.1-14.64)	1 2.08 (0.32-13.26)
Exposure to second-hand smoke from opium drug abuse				
Yes No	1 -	1 5 (1.28-19.44)	1 3.3 (0.44-24.5)	1 1.37 (0.46-4.07)
Contact with skin and hair of animals				
Yes No	1 -	1 0.79 (0.09-6.92)	1 0.37 (0.05-2.64)	1 1.59 (0.43-5.83)
The use of perfume or deodorant				
Yes No	1 -	1 0.75 (0.19-2.86)	1 1.18 (0.27-5.09)	1 0.7 (0.27-1.79)
Contact dust				
Yes No	1 -	1 2.79 (0.56-13.9)	1 -	1 6.76 (1.86-24.5)
Contact pollen and plant				
Yes No	1 -	1 2.79 (0.56-13.9)	1 0.22 (0.02-2.42)	1 3.31 (1.12-9.77)
Aspirin				
Yes No	1 -	1 -	1 -	1 3.17 (0.42-23.81)

## Discussion

In this study, the two groups were different in terms of education, but there was no significant difference between confounding variables such as age, work history, hookah smoking, family history of asthma and allergic rhinitis, contact with pets’ skin and hair, or use of aspirin in the two groups. However, variables such as smoking, drug abuse, contact with dust, use of perfume and air freshener, and contact with pollen and plants differed between the two groups. Clearly, differences in some of these variables are in relation to the nature of their jobs and their social and economic level. For example, an office employee would be expected to be more educated and use perfume more than a garden worker, while it is evident that a palm garden worker deals with dust and pollen more than an office employee. Therefore, it is likely that contracting asthma and/or allergic rhinitis in palm tree garden workers is related to their occupation.

In this study, we found no significant relationship between cigarettes and hookah smoking with either asthma or allergic rhinitis. Mehrabi et al in Kurdistan also found no correlation between smoking and asthma, but they observed a significant relationship between age and asthma (6). According to Liebhart et al, second-hand smoke was not a risk factor for asthma, while direct smoking was (7).

In this study, the target population was selected from people aged 20 years and older, with an average age of 38.47±10.26 years. The mean age of patients with asthma was 39±10.26 years. In our study, no relation was found between age and asthma. In the study conducted by Ayatollahi et al in Shiraz, no relation was seen between age and asthma either (8). In a study in Mashhad, Iran investigating the prevalence of asthma in the general population, the prevalence of asthma was 2.8% in adults, of whom 75% had been diagnosed with asthma previously. Further, the prevalence of asthma increased with age (9).

In a study by Mehrabi in Kurdistan in people aged 15 to 64 years, the prevalence of asthma was 2.3% (6). In a study in people aged 20 to 44 years in Uremia, Iran, the prevalence of asthma was 3.3% (10). In a study by Sadegh Niyat et al, the prevalence of asthma was highest in farmer and ranchers; while laboratory technicians and employment in the food industry were the most common job groups among asthmatic patients. The higher prevalence of asthma in farmers may be due to their occupational exposure to risk factors for asthma (11). Similarly, in this study, there was a significant relationship between working in palm tree gardens with asthma and allergic rhinitis.

In a study conducted by Pazuki et al in Tehran, sensitivity to outdoor inhaled allergens such as pollen was higher than other inhaled allergens (12). In Hamadan, the prevalence of allergic rhinitis was 17.7% among students aged 14–13 years (13); in Bushehr, the prevalence was 25.5%, and in Orumieh it was 23.6% among students aged 14–13 years (14,15). In Tehran, the prevalence of allergic rhinitis was 33.7% (16), and in Kashan it was 29.6%, among students (17). In the present study, the prevalence of the symptoms of allergic rhinitis such as sneezing, runny nose, or blocked nose was 34% among the palm tree garden workers. This is much higher than the other studies.

Studies have shown that parents’ smoking can increase allergic rhinitis in children (13). In the present study, no relationship was seen between allergic rhinitis with smoking or drug abuse. In the study conducted by Abbasi in Rasht, allergic rhinitis did not limit daily activities (18). Similarly, in our study, no significant relationship was found either.

The study conducted in 140 patients with allergic rhinitis in Malaysia showed that 85% of patients were sensitive to at least one inhaled allergen (19). A study conducted in 410 patients with allergic rhinitis in Ahvaz showed that outdoor allergens such as pollens were the most common (81%) allergic agent (20). In another study in 157 patients in Kerman, outdoor inhaled allergens with the prevalence of 55.9% were the most common allergens identified in that area, according to a skin prick test (21).

In a study conducted among ordinary people in Mashhad, the rate of allergic disorders was 27.5%. The rates for allergic rhinitis, atopic dermatitis, conjunctivitis, rhino-conjunctivitis, and asthma were 22.4%, 6.6%, 13.5%, 9.5%, and 2.3%, respectively. Half of the allergic people had a running nose while the nonallergic group had the other symptoms less frequently than allergic patients. Dust and pollen were the main allergens that were detected in this study (22).

In a study in 212 patients with allergic rhinitis in Shiraz, the rates of allergens were 92.4% for pollens, 22.7% for mites, and 8.3% for molds. The main pollens responsible for allergy in this research was weeds 92 (75.4%), grass 78 (63.9%), and trees 68 (55.7%) (23). In a study in Europe, Chatzi et al stated that the rate of asthma was 3.2% in farmers and 5.1% in flower and ornament plant producers. The result of their study showed that being a grape farmer in the Malevisi area in Greece is significantly correlated with allergic rhinitis and respiratory symptoms (24).

In the United Arab Emirates in 2005, Ahmed studied the allergenicity of date pollen in three gardens. Based on the distribution of pollen in the air and its distance from people, most exposure to date palm tree pollen occurred in March. People working in the date gardens had greater exposure to pollen than ordinary people, while date palm farms had higher concentrations of pollen when compared with the area near to the garden. The authors in this research suggested that keeping a distance of at least 200 m from date palm trees would decrease their symptoms dramatically. However, keeping in touch with high levels of pollen could possibly lead to allergy in workers who work in such fields (25).

However, in Saudi Arabia, Kawasaki et al. (1994) noted the fact that not all date palm tree pollens could cause an allergic reaction (26). In a study by Banner et al in Al-Ain, the United Arab Emirates, they showed that among allergic reactions to pollens in the air, date pollen caused the lowest percentage of allergies, compared with other pollens (27).

A limitation of this study was that we did not use the prick test or laboratory tests to confirm the existence of asthma or allergy. The main reason was a lack of cooperation and the difficulty of taking workers to specialized clinics in the city located far away from their working location.

## Conclusion

The prevalence of asthma and allergic rhinitis in palm tree garden workers seems to be higher than that in the general population. This occupational group should therefore undertake necessary precautions.
